# Wearable Sensors for Estimation of Parkinsonian Tremor Severity during Free Body Movements

**DOI:** 10.3390/s19194215

**Published:** 2019-09-28

**Authors:** Murtadha D. Hssayeni, Joohi Jimenez-Shahed, Michelle A. Burack, Behnaz Ghoraani

**Affiliations:** 1 Department of Computer and Electrical Engineering and Computer Science, Florida Atlantic University, Boca Raton, FL 33431, USA; mhssayeni2017@fau.edu; 2 Icahn School of Medicine at Mount Sinai, New York, NY 10029, USA; Joohi.Jimenez-shahed@mountsinai.org; 3 Department of Neurology, University of Rochester Medical Center, Rochester, NY 14642, USA; MichelleBurack@youcenteredhealth.org

**Keywords:** Parkinsonian tremor, continuous monitoring, wearable sensors, gradient tree boosting, deep learning, LSTM

## Abstract

Tremor is one of the main symptoms of Parkinson’s Disease (PD) that reduces the quality of life. Tremor is measured as part of the Unified Parkinson Disease Rating Scale (UPDRS) part III. However, the assessment is based on onsite physical examinations and does not fully represent the patients’ tremor experience in their day-to-day life. Our objective in this paper was to develop algorithms that, combined with wearable sensors, can estimate total Parkinsonian tremor as the patients performed a variety of free body movements. We developed two methods: an ensemble model based on gradient tree boosting and a deep learning model based on long short-term memory (LSTM) networks. The developed methods were assessed on gyroscope sensor data from 24 PD subjects. Our analysis demonstrated that the method based on gradient tree boosting provided a high correlation (*r* = 0.96 using held-out testing and *r* = 0.93 using subject-based, leave-one-out cross-validation) between the estimated and clinically assessed tremor subscores in comparison to the LSTM-based method with a moderate correlation (*r* = 0.84 using held-out testing and *r* = 0.77 using subject-based, leave-one-out cross-validation). These results indicate that our approach holds great promise in providing a full spectrum of the patients’ tremor from continuous monitoring of the subjects’ movement in their natural environment.

## 1. Introduction

Tremor is an involuntary movement disorder, common in many middle-aged and older adults. It is characterized by approximately rhythmic shaking of hands, head, trunk, voice, or legs [1]. Tremor reduces the quality of life by interrupting the subjects’ activities such as reading, writing, and eating. Tremor can be divided into “resting tremor”, which occurs as subjects relax their muscles and “action tremor”, which happens while the subjects perform voluntary muscle movements. There are two types of action tremors: postural and kinetic tremor. The postural tremor occurs when a person holds his/her hand(s) or leg(s) still, against gravity. The kinetic tremor happens when a person is engaged in different daily activities that involve voluntary movements of the muscles. There are several causes for tremor. Idiopathic Parkinson’s disease (PD) is the most common cause of resting tremor, although it may cause some action tremor as well [1].

A neurologist typically measures the severity degree of resting and action (postural and kinetic) tremor of PD patients during routine clinical visits. The neurologist performs the Unified Parkinson Disease Rating Scale (UPDRS) Part III assessment during which the patients are asked to perform three tasks: arm resting, arm extending, and touching the tip of their nose with their index finger (finger-to-nose testing) [2]. However, a single clinical examination often fails to capture the complete spectrum of tremor that a PD subject experiences in routine daily life [3]. Wearable sensors, combined with machine learning algorithms, can be used in a patient’s natural living environment to estimate the severity rating of tremor based on the way that it manifests itself in movement patterns [4,5,6,7,8,9,10,11,12].

However, the majority of the existing approaches are task-dependent, meaning that they require the subjects to perform the standardized tasks as in the UPDRS-III to elicit tremor [5,6,7,10,11,12]. Some of these methods reported a high correlation with the gold-standard tremor subscore measured by a neurologist [5,6]. The authors in Ref. [5] reported $r^{2}$ = 0.88 when estimating postural tremor, and the work in Ref. [6] reported a correlation of 0.98 when estimating the total PD tremor. However, these methods rely heavily on the patients’ active engagement in performing the UPDRS-III tasks several times throughout a day. As a result, they can provide only a “snapshot” representation of tremor versus its complete spectrum. There is a need for a “task-independent” approach that can estimate tremor sub-score continuously without requiring the patients to perform any specific tasks. However, the existing task-independent approaches have been able to provide moderate to good performance (correlation of 0.81–0.87) because of the limitations of the underlying algorithms to characterize patterns of tremor from patients’ free body movements [4,9].

Gradient tree boosting, an ensemble model of weak regression or decision trees, has been successful in a broad range of applications, especially after the refinements that were introduced by Chen and Guestrin to deal with its over-fitting problem and to make the model faster [13]. Recently, it has been successfully applied to predict PD motor impairments in a closed-loop control of the deep brain stimulation [14], to discriminate between healthy and patients with essential tremor [15], and to detect voice pathology in PD patients [16,17,18]. In addition to the traditional machine learning approaches, deep learning has also been recently used to estimate different aspects of PD-related motor function; for example, it was used for detection of response to medication in PD patients [19,20], and estimation of bradykinesia [21] and PD severity [22,23]. In a recent work, we used long short-term memory (LSTM) networks to estimate the total UPDRS-III score [23], which is different from the focus of the present paper.

In this paper, we developed two new task-independent approaches based on gradient tree boosting and LSTM networks to estimate tremor (resting and action) severity using wearable sensor data collected while patients performed a variety of daily living activities (ADL). We investigated the hypothesis that machine learning algorithms are able to utilize the resting intervals within ADL to track and quantify resting tremor, and also separate rhythmic shaking from normal ADL to estimate action tremor. Such methods do not require the patients to perform any standardized tasks so that they could provide a continuous measure of tremor severity that PD patients experience over a typical day and enable the treating neurologist to adjust the patients’ medications effectively [24,25,26].

## 2. Dataset

### 2.1. Data Collection

**Figure 1:**
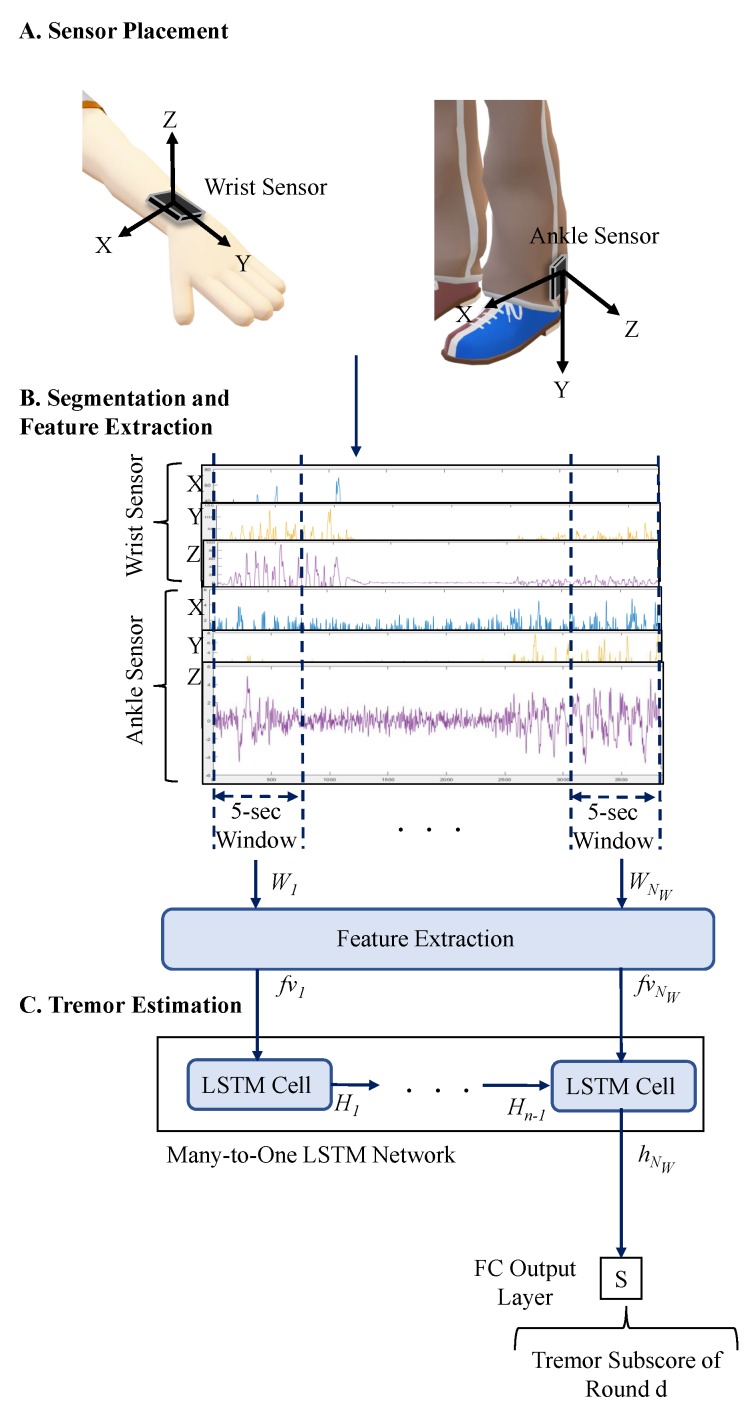
(**A**) Placement of the wearable sensors on wrist and ankle; (**B**) A total of 78 features are extracted from every 5-s segment of the data; (**C**) Estimation of the Parkinsonian tremor subscore based on the LSTM method. $N_{W}^{\left(d\right)}$ is the number of 5-s windows in round *d* and *H* represents the hidden states.

Twenty four PD subjects (58.9 ± 9.3 years old, 14 males) were recruited to record their motion data in two study protocols [9,27,28] approved by the Institutional Review Board at Rochester Medical Center and in accordance with the Helsinki Declaration. Prior to the experiments, the subjects provided written informed consent. In both protocols, the subjects stopped their medication the night before the experiment, and the experiment started in the morning. Yet, if the subjects were unable to withdraw their medication overnight, they came to the laboratory near the time of a scheduled dose of their PD medication. On the day of the experiment, one motion sensor (Great Lakes NeuroTechnologies Inc., Cleveland, OH, USA) was mounted on the wrist, and one on the ankle of the PD most-affected side of each subject to record the subjects’ body movements while performing a variety of ADL. The sensor placement is illustrated in Figure 1A. Each sensing unit consisted of triaxial gyroscope and accelerometer with a sampling frequency of 64 Hz.

Fifteen of the subjects were instructed to perform a round of specific ADL, such as walking, resting, eating, drinking, dressing, combing hair, putting groceries on a table, and cutting food [27,28]. The motion data was recorded only when they performed these activities. Next, these subjects took their routine PD medications to coincide with their morning dose. Later, they repeated the ADL at the beginning of every hour of the experiment up to four hours. Thus, up to four rounds of movement data were collected. Comprehensive UPDRS-III assessments were performed every hour of the experiment before each round of the ADL.

For the remaining nine subjects, the recording was performed continuously for the entire experiment, which lasted for approximately two hours [9]. At the beginning of the experiment, a comprehensive UPDRS-III assessment was performed. Next, the subjects were instructed to cycle through six stations in a home-like setting to perform different ADL, such as hygiene, dressing, eating, desk work, entertainment, and laundry. After cycling through all the stations at least once, these subjects took their routine PD medications. Later when the neurologist confirmed that the medications kicked in, they were asked to cycle through the stations again at least once. At the end of the experiment, another comprehensive UPDRS-III assessment was performed.

At least two comprehensive UPDRS-III assessments were performed for every subject during the experiment. The PD medication took between 33 to 104 min to kick in. Ten of the subjects were due for another scheduled dose of PD medication during the experiment. Nine of them took the medication before the third hour of the experiment, and one took it before the second hour of the experiment. Also, no booster dose was added to the first dose. The average duration of the disease for all the subjects was 9.9 ± 3.7 years, and the average Levodopa Equivalent Daily Dose (LEDD) was 1251 ± 468. The average UPDRS-III score and tremor subscore was 21.8 ± 12.1 and 1.6 ± 3, respectively.

### 2.2. Data Preprocessing

We used the total tremor subscore from the UPDRS-III assessment, which consists of five resting-tremor subscores of the head, left and right hand, and left and right leg as well as two action-tremor subscores from the left and right hand. The range of total tremor subscore is between 0 (no tremor) and 28 (severe tremor in all extremities). The tremor subscore may vary during a one-hour interval, so we only used the movement data that is closest to an UPDRS-III assessment. There were 2–4 UPDRS-III examinations per subject. We selected one round of activities after each UPDRS-III examinations for the 15 subjects for whom the data was recorded intermittently for the ADL. The maximum duration of each round for these subjects was 5 min. For the other nine subjects for whom the data was recorded continuously, we selected two consecutive rounds after the first UPDRS procedure and two consecutive rounds before the second UPDRS procedure if the duration of the recorded data was 20 min before and after the medications kicked in. The maximum duration of each round for these these subjects was 10 min. Note that the movement data recorded during the UPDRS-III assessment was excluded in order to eliminate the effect of the UPDRS-III specific tasks in our analysis. This process resulted in a total of 91 rounds of movement data. Table 1 shows the number of rounds and their total duration for each subject. Please note that the first 15 subjects in Table 1 performed the round of ADL and the last nine performed the continuous ADL. Each round was used as the input of our machine learning models. The associated tremor severity rating, as determined by the closest UPDRS-III assessment, was used as the predicted output of the models. The results reported by [4,6,12] showed that tremor predictive models performed better when gyroscopes data were used (vs. accelerometer data). Thus, only gyroscope signals were used in this work. A bandpass FIR filter with a 3 dB cutoff frequency between (0.5–15 Hz) was used to filter the movement data of each round to eliminate low and high-frequency noises.

## 3. Method

This section starts by describing our feature extraction technique followed by a description of the developed tremor severity estimation methods based on gradient tree boosting and LSTM.

### 3.1. Segmentation and Feature Extraction

In a recent study [29], we investigated how different signal features were associated with PD symptoms. Given that the focus of the present study is tremor estimation, we used the outcome of that study and extracted seventy eight features that were representative of tremor. To extract the features, the sensor data was segmented into non-overlapping five-second windows to represent the tremor patterns with oscillations of 4–6 Hz frequency [30,31]. The segmentation process is shown in Figure 1B. Next, we extracted the features listed in Table 2 from each segment. The details of the extracted features are provided in Ref. [29]. The extracted feature vectors ($\vec{fv}\in R^{N_{Tr}}$), where $N_{Tr}=$ 78 is the number of features per segment, were organized for all the rounds as $D_{Tremor}={\left\{\left(F^{\left(d\right)},y^{\left(d\right)}\right)\right\}}_{r}^{N_{R}}$ with $(F^{\left(d\right)}\in R^{N_{W}^{\left(d\right)}\times N_{Tr}}$, $y^{\left(d\right)}\in R)$. $F^{\left(d\right)}=\left[{\vec{fv}}_{1}{\vec{fv}}_{2}\ldots {\vec{fv}}_{t}\ldots {\vec{fv}}_{N_{W}^{\left(d\right)}}\right]$, $N_{W}^{\left(d\right)}$ is the number of 5-s windows in round *d*, and $N_{R}=91$ is the total number of rounds.

### 3.2. Tremor Estimation Methods

#### 3.2.1. Gradient Tree Boosting

The gradient tree boosting algorithm [13] was used to assign a tremor subscore $y^{\left(d\right)}$ to its corresponding extracted feature vectors $F^{\left(d\right)}$. First, each ${\vec{fv}}_{t}^{\left(d\right)}$ in round *d* was mapped to the tremor subscore $y^{\left(d\right)}$. Using an ensemble of $N_{t}$ weak regression trees (${\left\{f_{i}\right\}}_{i=1}^{N_{t}}$), the method estimates the output $\hat{y}$ according to the function in Equation (1).

(1) $${\hat{y}}_{t}^{\left(d\right)}=\sum_{i=1}^{N_{t}}f_{i}\left({\vec{fv}}_{t}^{\left(d\right)}\right)$$

where $f_{i}\left({\vec{fv}}_{t}\right)=w_{q({\vec{fv}}_{t})}$ is the space of regression tree *i* with *L* leaves, $q({\vec{fv}}_{t})$ is the structure of the tree that maps ${\vec{fv}}_{t}$ to an index and represents the corresponding tree leaf, and $w\in R^{L}$ is the leaf weights. Learning the regression trees is performed using the additive training strategy. In this strategy, one tree is learned at each iteration by minimizing an objective function. This function includes the first and second gradient statistics of a loss function based on the difference between the estimated tremor subscore ${\hat{y}}_{t}^{\left(d\right)}$ and the tremor subscore $y^{\left(d\right)}$ of round *d*. The trained model was used to estimate the tremor subscore of all the feature vectors in one round. Then, the average of the estimated tremor subscores in one round was calculated and used as the estimated tremor subscore of each round ${\hat{y}}^{\left(d\right)}$.

#### 3.2.2. Deep Learning Model

A deep learning model based on LSTM was developed to associate the feature vectors of each round $F^{\left(d\right)}$ to the round’s tremor score $y^{\left(d\right)}$. LSTM is a recurrent neural network that we use to learn the temporal dependencies in time series of the motion signals. LSTM cell consists of four gates: input (*i*), input modulation (*g*), forget (*f*) and output (*o*), and a memory cell ($c_{t}$ at time step *t*). Each of the gates has specific functionality by processing a feature vector ${\vec{x}}_{t}$ at time *t* using $N_{H}$ hidden states ($h_{t-1}\in R^{N_{H}}$) and $N_{H}$ internal states ($c_{t-1}\in R^{N_{H}}$) from the time step t−1. Mathematically, the gates’ functionality is described below:

(2) $$\begin{array}{c} i_{t}=\sigma \left(W_{xi}{\vec{x}}_{t}+W_{hi}h_{t-1}+b_{i}\right) \end{array}$$

(3) $$\begin{array}{c} g_{t}=\phi \left(W_{xg}{\vec{x}}_{t}+W_{hg}h_{t-1}+b_{g}\right) \end{array}$$

(4) $$\begin{array}{c} f_{t}=\sigma \left(W_{xf}{\vec{x}}_{t}+W_{hf}h_{t-1}+b_{f}\right) \end{array}$$

(5) $$\begin{array}{c} o_{t}=\sigma \left(W_{xo}{\vec{x}}_{t}+W_{ho}h_{t-1}+b_{0}\right) \end{array}$$

(6) $$\begin{array}{c} c_{t}=f_{t}c_{t-1}+i_{t}g_{t} \end{array}$$

(7) $$\begin{array}{c} h_{t}=o_{t}\phi \left(c_{t}\right) \end{array}$$

where σ is the logistic sigmoid activation function, ϕ represents the tanh activation function and $W_{ab}$ is the weight matrix of an LSTM gate with the input or hidden states (a={x,h} and b={i,g,f,o}). In this work, we used the LSTM design in Ref. [32], which has been commonly used in signal processing applications.

Depending on the LSTM design, the number of extracted feature attributes $N_{Tr}$ and the number of the LSTM’s hidden states $N_{H}$ could be different. So, the first step is to transform each feature vector (${\vec{fv}}_{t}^{\left(d\right)}$) linearly to a new feature vector ${\vec{x}}_{t}^{\left(d\right)}$ with the same length as the number of the LSTM’s hidden states $N_{H}$ using Equation (8).

(8) $${\vec{x}}_{t}^{\left(d\right)}=W_{fx}{\vec{fv}}_{t}^{\left(d\right)}+b_{fx}$$

where $W_{fx}$ and ${\vec{b}}_{fx}$ are a weight matrix and bias vector, respectively. Next, the transformed feature vector ${\vec{x}}_{t}^{\left(d\right)}$ is passed to a many-to-one LSTM network to estimate a tremor subscore (${\hat{y}}^{\left(d\right)}$). This network processes a single feature vector representing one segment at a time and continues to the last feature vector ${\vec{x}}_{N_{W}}^{\left(d\right)}$ in a given round. The hidden states $h_{N_{W}}^{\left(d\right)}$ from processing ${\vec{x}}_{N_{W}}^{\left(d\right)}$ was passed through a fully connected layer to estimate the tremor subscore of round r (${\hat{y}}^{\left(d\right)}$) as shown in Equation (9). The overall algorithm is shown in Figure 1. The optimization of the LSTM hyperparameters, including the number of layers and hidden states as well as the learning and dropout rates, are provided in the Results Section.

(9) $${\hat{y}}^{\left(d\right)}=W_{hy}h_{N_{W}}^{\left(d\right)}+b_{y}$$

where $W_{hy}$ and ${\vec{b}}_{y}$ are a weight matrix and bias vector, respectively.

## 4. Results

The extracted features were used to train and test the two developed models on the data of 24 PD subjects described in Section 2. We used a *held-out testing* approach to investigate the models’ efficacy in estimating tremor subscores. The data from the 15 subjects who performed rounds of specific ADL was used to train the models, and the data from the remaining nine subjects who performed continuous ADL were held out for testing the models. This data split resulted in using approximately 64% of the data for training and 36% for held-out testing. A leave-one-subject-out cross-validation method was used on the training data to optimize the model’s hyper-parameters. The mean and standard deviation of the training data were calculated and used to normalize the entire data. Pearson Correlation (*r*) and mean absolute error (MAE) were used to evaluate the developed methods by comparing the algorithm-predicted tremor subscore $\hat{y}$ and the one from the clinical examinations *y*. The testing *r* and MAE were reported for the estimated tremor subscores of the held-out data. Two experiments were performed. The first experiment compared the two models for estimating the total tremor subscore. The method with the highest performance was used in the second experiment to estimate the resting and action tremor subscores separately.

We repeated the above training–testing approach using a *leave-one-subject-out* cross-validation testing to investigate whether the models can be generalized to new subjects. There are 24 subjects in the dataset, so we performed 24 cross-validation iterations. At each cross-validation iteration, the data of one subject (one fold) was held-out and used for testing of the developed method. The data of the remaining 23 subjects (23 folds) were divided into 80% for training and 20% for validation purposes to optimize the model’s hyper-parameters. Please see Supplement Figure S1 of the Supplementary Materials for a visual description of the performed cross-validation. Using this method, the average duration of the test data is 22 min for all the cross-validation iterations.

### 4.1. Total Tremor Subscore Estimation

We used the the gradient tree boosting and LSTM algorithms to estimate the total tremor subscore (ranged between 0–28). The gradient tree boosting algorithm was implemented using XGboost library [13]. The learning rate was 0.1. A grid search was applied to find the optimal number of regression trees in the range of 10–200 with a step of 20. The tree depth was in the range of 3–10 with a step of 2. The percentage of used-features per tree was in the range of 10% to 50% with a step of 10%. For the held-out testing, an ensemble of 30 regression trees resulted in the highest validation correlation (*r* = 0.98 (*p* < $1\times 10^{-4}$)). Evaluating this model on the held-out set resulted in 1.56 MAE and *r* = 0.96 (*p* < $1\times 10^{-4}$). We repeated this process for the leave-one-subject-out testing. The best validation results were 1.02 MAE and *r* = 0.96 (*p* < $1\times 10^{-4}$) when ensemble of 170 regression trees was used. The testing correlation was (*r* = 0.93 (*p* < $1\times 10^{-4}$)), and the testing MAE was 1.18.

The LSTM network was implemented in Python using TensorFlow library [33]. The network weighs were initialized using random values from a normal distribution and trained for 200 epochs using Adam optimizer [34]. To prevent the LSTM network from memorizing the training sequences, the data was augmented. The augmentation was performed by clipping each round of feature vectors with a random start point. In addition, a dropout of 0.3 after each LSTM layer with a mini-batch of size 4 and norm-2 regularization were applied in order to reduce overfitting. In each mini-batch, the feature vectors of the selected rounds were repeated so that all the rounds have the same length as the longest round. We used an LSTM network with 3 layers as shown to be suitable for movement data representations of PD subjects [23]. A grid search was performed on a 3-layer LSTM network to find the optimal number of hidden states (16–224). Learning rate of $1\times 10^{-3}$ was used during the training. After each epoch, the model was evaluated on the validation data, and the model with the maximum value of correlation *r* was used on the testing data. For the held-out testing, a 3-layer LSTM with 160 hidden states resulted in the highest validation correlation (*r* = 0.94 (*p* < $1\times 10^{-4}$)), and evaluating this model on the held-out set resulted in 1.33 MAE and *r* = 0.84 (*p* < $1\times 10^{-4}$). For the leave-one-subject-out testing, the maximum validation correlation was 0.89 (*p* = 0.019) when a 3-layer LSTM with 224 hidden states was used. The testing correlation and MAE of this network were *r* = 0.77 (*p* < $1\times 10^{-4}$) and 1.32, respectively.

**Figure 2:**
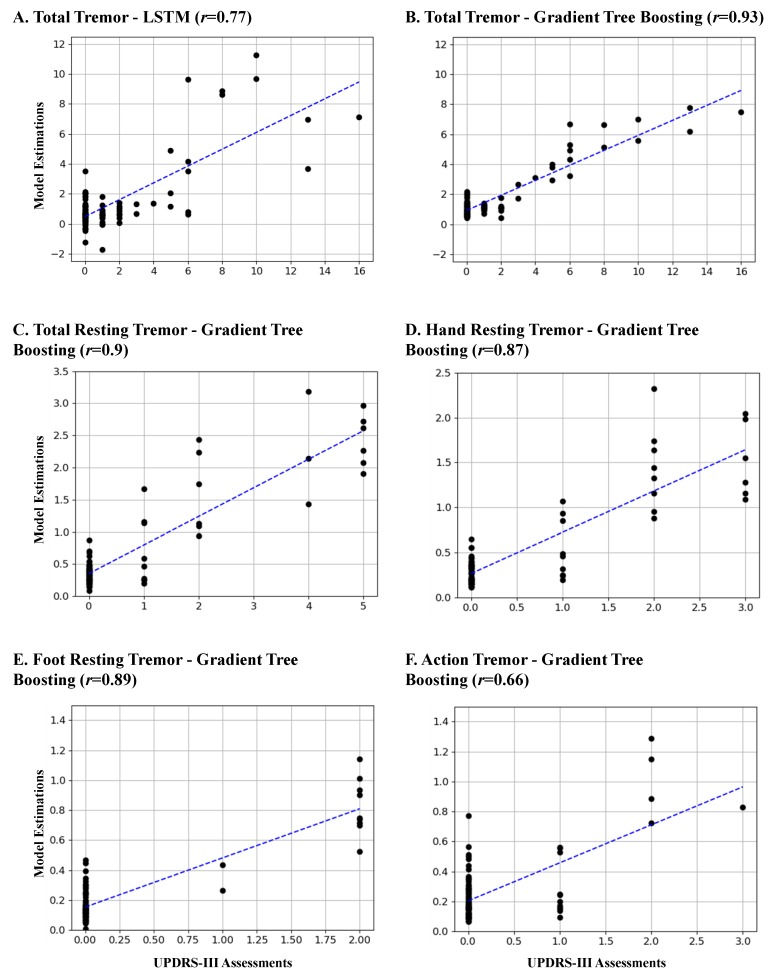
The estimated tremor subscores versus the values estimated from the UPDRS-III assessment. (**A**) The total tremor subscores using the LSTM method; (**B**) The total tremor subscores using the gradient tree boosting method; The estimations using the gradient tree boosting for (**C**) total resting tremor; (**D**) hand resting tremor; (**E**) foot resting tremor; and (**F**) action tremor. The blue dashed line indicates the fitted line to the data.

A list of the results of the held-out and leave-one-subject-out testing is provided in Table 3. Two main observations can be made from this table. The first observation is that the gradient tree boosting method outperforms the LSTM-based approach. The reason for such a behavior could be that the deep learning models typically perform better for larger datasets. The second observation is that the leave-one-subject-out testing performs a more rigorous evaluation of the methods compared to the held-out testing approach. Hence, we continued our analysis using the gradient tree boosting method and leave-one-subject-out testing. Figure 2A shows the estimated tremor subscores against the subscores from the UPDRS-III assessments.

An experiment was performed to investigate whether enforcing an overlap between the segmentation windows will improve the performance of the gradient tree boosting method. The same procedure was repeated except for using a 50% overlap between the sliding 5-s windows. The best validation performance was *r* = 0.96 (*p* < $1\times 10^{-4}$) when an ensemble of 90 regression trees was used. The testing correlation was (*r* = 0.93 (*p* < $1\times 10^{-4}$)), which was the same correlation as with no overlapping. This behavior was expected as the method reports the average of the estimated tremor subscores of all the windows within every round, which could be up to 10-mins long, as the estimated tremor subscore of each round. So, there is no need for any overlapping to make sure that the method picks up the tremor in the windows within each round. Given that the 50% overlapping is computationally more expensive by doubling the number of extracted feature vectors and does not provide any improvement, we continued the remaining analysis with no overlapping.

**Figure 3:**
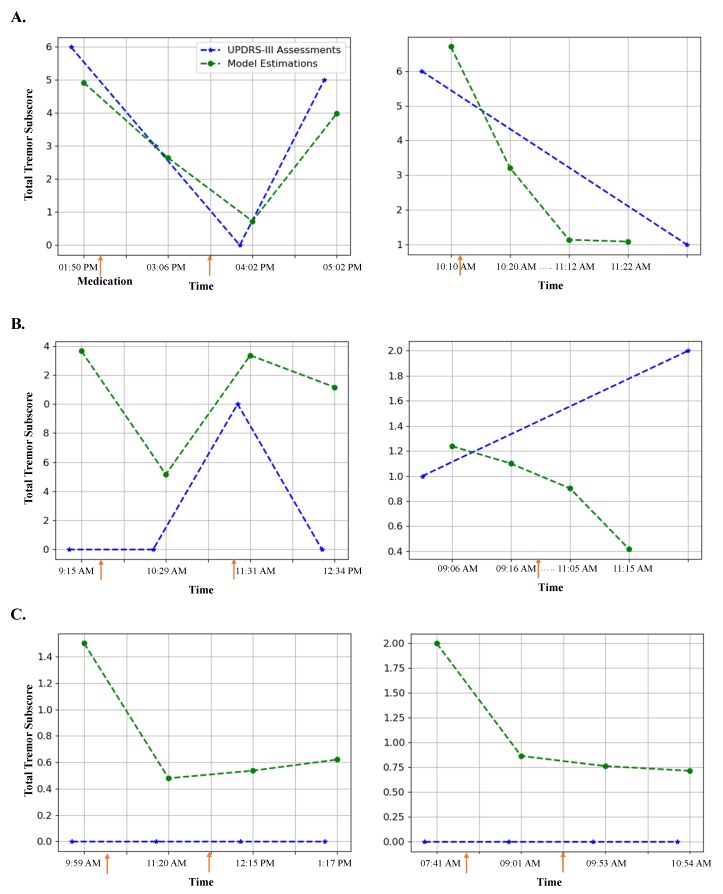
Examples of total tremor estimation over time using the gradient tree boosting model in comparison with the estimation from the UPDRS-III assessment. (**A**) Two examples with high correlation; (**B**) Two examples with moderate to low correlation; (**C**) Two examples from non-tremor-dominant subjects. The PD medication intake time is denoted as an orange arrow. Note that in cases of the subjects in the right-hand side of A-B, only two UPDRS-III assessments were performed while four tremor estimations were performed using our developed model.

We also investigated if the method performs differently for different genders. The gradient tree boosting method performed approximately the same for both genders with *r* = 0.94 (*p* < $1\times 10^{-4}$) and MAE = 1.23 for male subjects and *r* = 0.91 (*p* < $1\times 10^{-4}$) and MAE = 0.9 for female subjects. The estimated total tremor subscore using the gradient tree boosting method for six of the subjects are shown in Figure 3. In this figure, panel A and B are from the *tremor-dominant subjects* who had a tremor subscore >=1, and panel C is from the *non-tremor-dominant subjects* who had a tremor subscore of 0 during all the UPDRS-III assessments. Panel A shows two cases where the method provides a good match with the UPDRS-III total tremor subscore, while Panel B shows two cases that the predicted values did not match the exact values from the UPDRS-III report.

**Figure 4:**
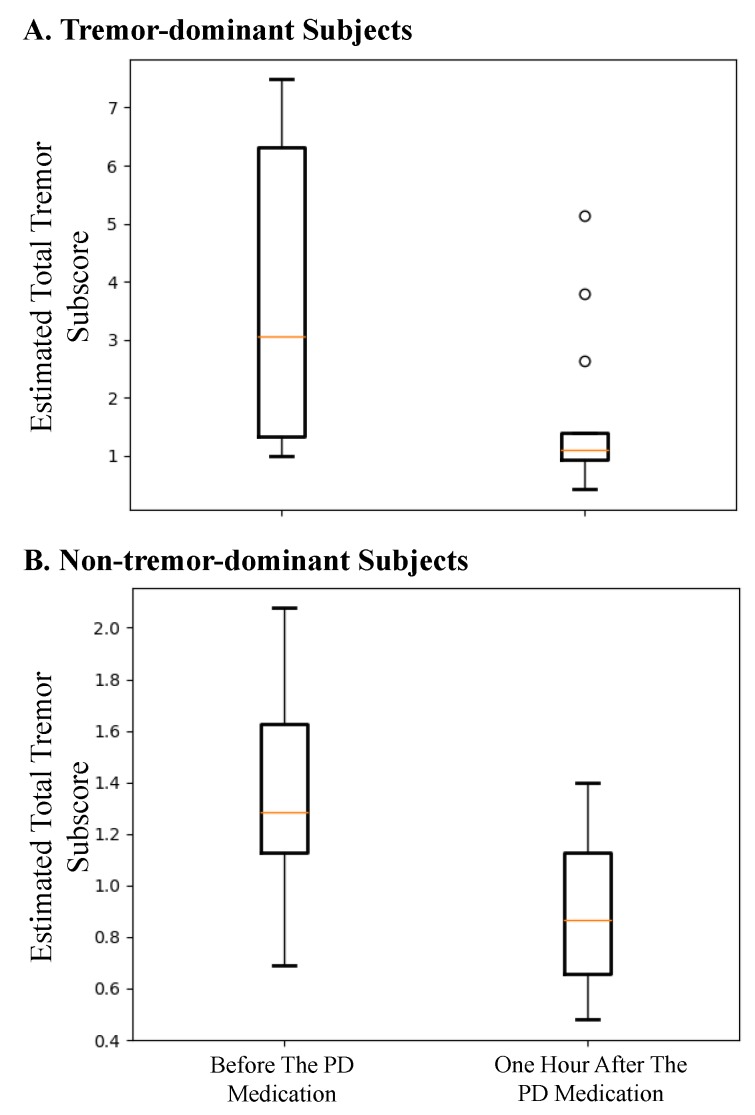
Total tremor subscore estimated using the gradient tree boosting method for (**A**) tremor-dominant and (**B**) non-tremor-dominant subjects before and about one hour after taking the first scheduled dose of PD medication.

Next, we investigated the method’s ability to estimate the change in the presence of tremor before and after taking the medication. The box plots in Figure 4 show the model-estimated total tremor before and approximately one hour after taking the medication for the tremor-dominant and non-tremor-dominant subjects. As can be seen, the method shows a statistically significant reduction (*p* < 0.02) in tremor severity with taking the medication for both groups of subjects.

Lastly, we investigated whether the activities that resemble the tasks in the UPDRS-III assessment affect the ability of the algorithm in detecting the tremor subscore. For this purpose, we identified the data intervals where the subjects were resting. An example of such intervals was resting while watching TV. Then, we repeated the experiment after removing these resting intervals from the testing phase. This analysis resulted in a correlation of *r* = 0.91 (*p* < $1\times 10^{-4}$) and the testing MAE of 1.15, which do not show a significant decrease in performance, indicating that the algorithm can reliability estimate tremor from free-body movements and not just the activities that resemble the tasks in the UPDRS III.

### 4.2. Resting and Action Tremor Subscore Estimation

The ensemble model based on gradient tree boosting was trained and tested to estimate resting and action tremor separately. For the resting tremor, three models were trained to estimate the total resting tremor on both hand and foot, only hand resting tremor, and only foot resting tremor. The same datasets with all the ADL were used. However, we used only the wrist sensor to estimate the hand resting and action tremor and only ankle sensor to estimate the foot resting tremor. Also, instead of using the total resting and action tremor on all the extremities as in the previous experiment, we used different combinations of the tremor subscores. Training the model for the resting tremor of both hand and foot was performed using the summation of the hand and foot resting tremor (range 0–8) from the UPDRS-III assessments. Only hand resting tremor (range 0–4) and only foot resting tremor (range 0–4) were also used to train and test the models for the only hand resting tremor and only foot resting tremor, respectively. For the action tremor, only one model was trained and evaluated using the action tremor item in the UPDRS-III assessment.

The same procedure as in the previous experiment was repeated to select the hyperparameters of the gradient tree boosting and train three resting tremor models and one action tremor model. The results are reported in Table 3. As shown, the method provided a correlation of *r* > 0.87 (*p* < $1\times 10^{-4}$) on the testing data for all the models except the one that was used to estimate the action tremor. The action tremor model resulted in r= 0.66 (*p* < $1\times 10^{-4}$). Figure 2C–F show the estimated tremor using these four models against the tremor values from the UPDRS-III assessments. Lastly, we investigated whether the LSTM method could provide better performance for the action tremor. So, we trained an LSTM-based model to estimate action tremor. The LSTM network yielded lower testing results with *r* = 0.43 (*p* < $1\times 10^{-4}$) and MAE = 0.4.

### 4.3. Feature Analysis

**Figure 5:**
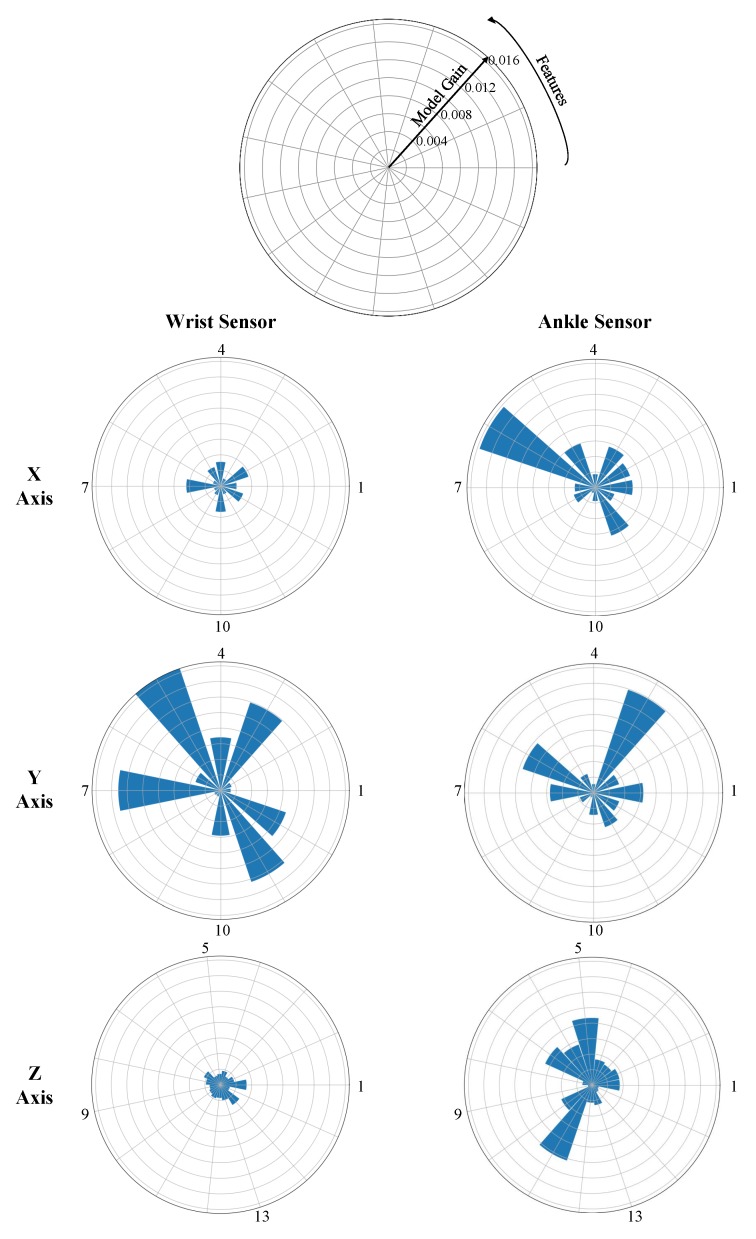
Feature importance based on the ensemble model gain. Each radar plot represents the gain using the features extracted from specific axis and specific sensor. The numbers correspond to each of the features (refer to Table 2).

We investigated the feature importance to the total tremor estimation. For this purpose, the gain of each feature for estimating the tremor subscore in the gradient tree boosting model was found. To visualize the features’ importance, we created a radar plot as shown in Figure 5. Each feature of Table 2 is shown on one spoke. We created six radar plots representing the features extracted from the X, Y, and Z axes of the wrist and ankle sensor. Please note that the features are placed counterclockwise, and the cross-correlation features of 13, 14, and 15 are shown on the Z-axis plots.

Several observations can be made from these plots. First, the features extracted from the Y signal of the wrist sensor resulted in a higher estimation gain. This means that the hand tremor occurred mainly around the Y axis. Second, the important features of the ankle sensor were not specific to an axis. As can be seen form Figure 5, the important features were spread on all the axes indicating that foot tremor was not manifested over a specific axis. Another interesting observation was that the feature of 4–6 Hz signal power was not selected as an important feature from the wrist, while the percentage power of frequencies >4 Hz was selected. This indicates that the tremor frequency was greater than 6 Hz. Finally, we identified the important features with a gain of more than 0.5% and listed them for the wrist and ankle sensors in Table 4.

## 5. Discussion

Wearable sensors can be used to provide an objective estimation of Parkinsonian tremor from continuous movements of patients in their natural living environment. In this paper, we investigated the application of two machine learning algorithms based on gradient tree boosting and LSTM-based deep learning to make the estimation using data from two gyroscope sensors placed on the upper and lower extremity of the patients with PD. Our analysis indicated that the gradient tree boosting method was able to estimate the total tremor as well as the resting tremor subscore with high accuracy (see Table 3). The LSTM-based method provided lower performance. The method’s validation loss saturated at 1.5, whereas the training loss was close to zero. This indicates the LSTM method overfitted the training data even with the precautions that were taken in the training phase (i.e., 0.3 dropout layers, mini-batch of size 4 and norm-2 regularization).

The gradient tree boosting method was able to estimate the same tremor subscore values as estimated from the UPDRS-III assessments in most cases. The left plot of Figure 3A illustrates a case that the method provides a close match. In the right plot of Figure 3A, although there were only one UPDRS-III assessment at the beginning and one at the end, our method showed the decline in tremor severity with a high temporal resolution throughout the experiment.

Another interesting observation is that our method was able to show the decline in tremor after taking the medication even in cases where our results did not match the total tremor subscores from the UPDRS-III assessments. For example, in the left plot of Figure 3B, the method shows a decline from 1.4 before medication to 0.5 after taking the medication although the UPDRS-III estimation of total tremor was 0 tremor for both cases. This discrepancy could be because we used the data after the assessment was performed and the tremor could have appeared later; or it could be because of the subjectivity of the tremor assessment during the UPDRS-III assessments [35,36]. In the right plot of Figure 3B, the UPDRS-III estimation of tremor indicates an increase in tremor while our method shows a decline after taking medication. Such a behavior is also evidenced in Figure 4, where there is a statistically significant reduction (*p* < 0.02) in the estimated tremor from before to after taking the medication.

We observed that the model results in a lower specificity for cases with non-tremor-dominant subjects. For example in Figure 3C, no tremor was reported throughout the experiment based on the UPDRS-III examinations, but our method detected some level of tremor. This is consistent with previous work for automated tremor estimation [5]. However, it is interesting that our method still shows a decline in tremor with taking the medication. In Figure 3C of the left plot, the method also shows a slight increase in the estimated tremor as time passes from the medication.

Although the method provides high performance for the total tremor estimation, its performance is not evenly distributed for all the tremor subscores. Our investigations indicates that the performance is better for cases with a lower tremor subscore than the ones with a higher tremor subscore. As it can be seen in Figure 2, there were only seven cases with a total tremor subscore >6. This could be the reason for the model not being effectively trained to estimate those cases. Increasing the number of subjects with a higher tremor subscore is expected to improve the method’s performance.

Another observation is that as shown in the plots C-D of Figure 2, estimation of the resting tremor subscores was highly correlated with the UPDRS-III estimations on both hand and leg, whereas the estimation of action tremor subscores had a moderate correlation. Giuffrida and colleagues also reported a moderate correlation for the action tremor estimation even though they used only standardized tasks [5]. Please note that our method was able to successfully detect hand and leg tremor using only one sensor on the wrist and ankle, respectively.

Our analyses supported our hypothesis that machine learning methods could accurately estimate tremor from ADL. To make sure of this, we only included the data from free body movements during ADL and excluded any data during the UPDRS-III assessments from our analysis. In addition, in one experiment, we excluded any resting intervals with a duration of longer than 15 s from our analysis to exclude any data that resembles the UPDRS-III tasks. This analysis resulted in high testing correlation *r* = 0.91 (*p* < $1\times 10^{-4}$) and MAE = 1.15, which are slightly lower than when resting intervals were included. This performance indicates the competency of our method to estimate tremor from free body movements.

### 5.1. Comparison to Other Studies

Our method provided the highest performance among some of the UPDRS-III task-dependent methods and all the task-independent tremor estimation methods reported in the literature. For example, the task-dependent method in Ref. [7] resulted in *r* = 0.74 for hand resting tremor. The work in [10] provided *r* = 0.65 for resting and posture tremor, and the method in [12] provided *r* = 0.81 for resting tremor. Regarding the task-independent methods, Salarian and colleagues used the data from two sensors on each forearms to estimate total tremor subsocre [4]. In this method, the authors used an autoregressive-based model and estimated a tremor subscore for every 45-min interval of the data. They reported a Pearson correlation of *r* = 0.87 (*p* < 0.001) between the estimated and total tremor subscores. In comparison to this method, ours provided higher performance (*r* = 0.93 (*p* < $1\times 10^{-4}$)) over a shorter duration of data (every 10 min). Using a held-out testing approach, our method achieved even higher performance (*r* = 0.96 (*p* < $1\times 10^{-4}$)). This is important as our method is able to provide a better temporal resolution for the tremor estimation to provide a measure of the full spectrum of tremor as changes over time. In addition, their work used two sensors on the upper extremities, while we used only one sensor on wrist and were able to provide a high correction (*r* = 0.90 (*p* < $1\times 10^{-4}$)). The other task-independent work was reported by Pulliam and colleagues [9]. They used data from two gyroscope sensors mounted on the wrist and ankle [9] to quantify tremor, bradykinesia and dyskinesia in 12-s windows. However, they reported only the correlation between their models and the total UPDRS-III score, which was *r* = 0.81 over 10-min data intervals.

### 5.2. Study Limitations and Future Work

We used the data recorded from 24 subjects with PD as they performed ADL at a clinical setting to train and assess our approach. Our results indicated the potential of the developed algorithm for providing an estimate of tremor severity as the PD subjects experience over a typical day. Validation of our method for longer duration of test data and large cohort of subjects and in a home setting scenario with more complex activities requires further investigations. In this study, we estimated the resting and action tremor separately as provided in the UPDRS-III assessment. Estimation of the postural and kinetic tremor as provided in the Movement Disorders Society UPDRS Part III (MDS-UPDRS III) [2] assessment constitutes our future work.

## 6. Conclusions

We developed a machine learning algorithm to automatically estimate tremor severity from subjects’ free body movements. Our method consisted of a feature extraction and regression model. The former extracted 78 features from every 5-s segment of data while the latter estimated a tremor subscore for every 10-min duration of the data. Two regression models were considered in this study. One model used gradient tree boosting and the other was based on LSTM deep learning. The developed models were assessed using data from 24 PD subjects who had one wearable sensor on their most affected wrist and one on the most affected ankle as performed a variety of free body movements. Our evaluations demonstrated that gradient tree boosting resulted in the highest correlation (*r* = 0.96 with held-out testing and *r* = 0.93 using leave-one-out cross-validation) reported in the literature when using unconstrained body movements’ data. In addition, we investigated the potential of the gradient tree boosting model in estimating resting and action tremor. The model estimated resting tremor with a high correlation to the UPDRS-III assessment while action tremor was estimated with a moderate correlation. These results support that the developed tremor estimation method, combined with wearable sensors, provide a reliable approach for estimating tremor severity as PD subjects experience in a typical day, thereby providing necessary information for effective therapeutic management of patients suffering from Parkinsonian tremor.

## Figures and Tables

**Table 1:** Number of rounds and total duration of the movement data used for each subject.

Subject #	Number of Rounds	Total Duration (min)	Subject #	Number of Rounds	Total Duration (min)
**1**	4	12.20	**13**	4	14.28
**2**	4	13.42	**14**	4	15.97
**3**	4	14.38	**15**	4	10.61
**4**	4	13.86	**16**	4	40.00
**5**	4	14.95	**17**	4	37.92
**6**	4	13.26	**18**	4	40.00
**7**	3	10.33	**19**	3	26.60
**8**	3	10.69	**20**	4	40.00
**9**	4	14.30	**21**	4	40.00
**10**	4	13.68	**22**	4	40.00
**11**	4	15.62	**23**	2	20.00
**12**	4	13.86	**24**	4	40.00

**Table 2:** The extracted features in this work.

Feature Name	Used Signals	# of Features
1—4–6 Hz signal power	X, Y, Z—wrist and ankle	6
2—0.5–15 Hz signal power	X, Y, Z—wrist and ankle	6
3—Percentage power of frequencies >4 Hz	X, Y, Z—wrist and ankle	6
4—Number of autocorrelation peaks	X, Y, Z—wrist and ankle	6
5—Sum of autocorrelation peaks	X, Y, Z—wrist and ankle	6
6—Amplitude of the first autocorrelation peak	X, Y, Z—wrist and ankle	6
7—Lag of the first autocorrelation peak	X, Y, Z—wrist and ankle	6
8—Spectral entropy	X, Y, Z—wrist and ankle	6
9—First dominant frequency	X, Y, Z—wrist and ankle	6
10—Power of first dominant frequency	X, Y, Z—wrist and ankle	6
11—Second dominant frequency	X, Y, Z—wrist and ankle	6
12—Power of second dominant frequency	X, Y, Z—wrist and ankle	6
13—Cross-correlation	X and Y—wrist and ankle	2
14—Cross-correlation	X and Z—wrist and ankle	2
15—Cross-correlation	Y and Z—wrist and ankle	2
**Total Number of Features**		78

**Table 3:** Validation and testing results using data from 24 PD subjects.

Tremor Type	Sensor Used	Method Used (Specifications)	Held-Out Testing		Leave-One-Out Testing	
			MAE	r (p)	MAE	r (p)
Total rest and action tremor	Wrist and ankle	LSTM	1.33	0.84 (<$1\times 10^{-4}$)	1.32	0.77 (<$1\times 10^{-4}$)
Total rest and action tremor	Wrist and ankle	Gradient tree boosting	1.56	0.96 (<$1\times 10^{-4}$)	1.18	0.93 (<$1\times 10^{-4}$)
Total rest tremor	Wrist and ankle	Gradient tree boosting	1.20	0.94 (<$1\times 10^{-4}$)	0.58	0.90 (<$1\times 10^{-4}$)
Hand rest tremor	Wrist	Gradient tree boosting	0.76	0.91 (<$1\times 10^{-4}$)	0.41	0.87 (<$1\times 10^{-4}$)
Foot rest tremor	Ankle	Gradient tree boosting	0.46	0.92 (<$1\times 10^{-4}$)	0.27	0.89 (<$1\times 10^{-4}$)
Action tremor	Wrist	Gradient tree boosting	0.41	0.75 (<$1\times 10^{-4}$)	0.33	0.66 (<$1\times 10^{-4}$)

**Table 4:** Important features with a gain of greater than 0.5% in estimating the total tremor subscore.

Wrist Sensor		Ankle Sensor	
Important Features	Axis	Important Features	Axis
Feature #5: sum of autocorrelation peaks	Y	Feature #6: amplitude of the first autocorrelation peak	X, Y and Z
Feature #7: lag of the first autocorrelation peak	Y	Feature #3: percentage power of frequencies > 4 Hz	X and Z
Feature #11: second dominant frequency	Y	Feature #11: second dominant frequency	X and Z
Feature #3: percentage power of frequencies > 4 Hz	Y	Feature #5: sum of autocorrelation peaks	X and Y
Feature #12: power of second dominant frequency	Y	Feature #7: lag of the first autocorrelation peak	Y
Feature #4: number of autocorrelation peaks	Y	Feature #1: 4–6 Hz signal power	Y
Feature #10: Power of first dominant frequency	Y		

## Supplementary Materials

The following are available online at https://www.mdpi.com/1424-8220/19/19/4215/s1, Figure S1: A. Schematic of the leave-one-subject-out cross-validation. B. Data split for training a model and hyperparameters’ adjustments at every iteration.
