# Supplementary material for: Wearable Sensors for Estimation of Parkinsonian Tremor Severity during Free Body Movements
Source: Sensors (Basel). 2019 Sep 28;19(19):4215. doi: 10.3390/s19194215 (PMC6806340; doi:10.3390/s19194215)
Supplement: Supplementary file 1 [file sensors-19-04215-s001.pdf]

Supplementary Online Content for "Wearable Sensors for Estimation of Parkinsonian Tremor Severity During Free Body Movements"

One-Subject-Out Cross-Validation

The performance of a predictive model is overestimated when simply determined on the sample of subjects that was used to construct the model [1]. Hence, leave-one-subject-out cross-validation leaves the data of one subject out for testing and to identify whether the algorithm is generalizable to a new subject. We described the leave-one-subject-out cross validation as was used in our work in **Supplement Figure 1**. As shown in Figure 1A, the data of one patient is left out as "testing data" and the remaining data is used as "training and validation data". The data split for "training and validation data" at each iteration is shown in Figure 1B. At every iteration, a model is trained and optimized using the "training and validation data". Then, the trained model is tested on the "test data" to identifies the model's testing performance. This process was repeated for all the 24 subjects, and the average performance was reported in the paper. As shown in this diagram, there is no overlap between the training and testing data.

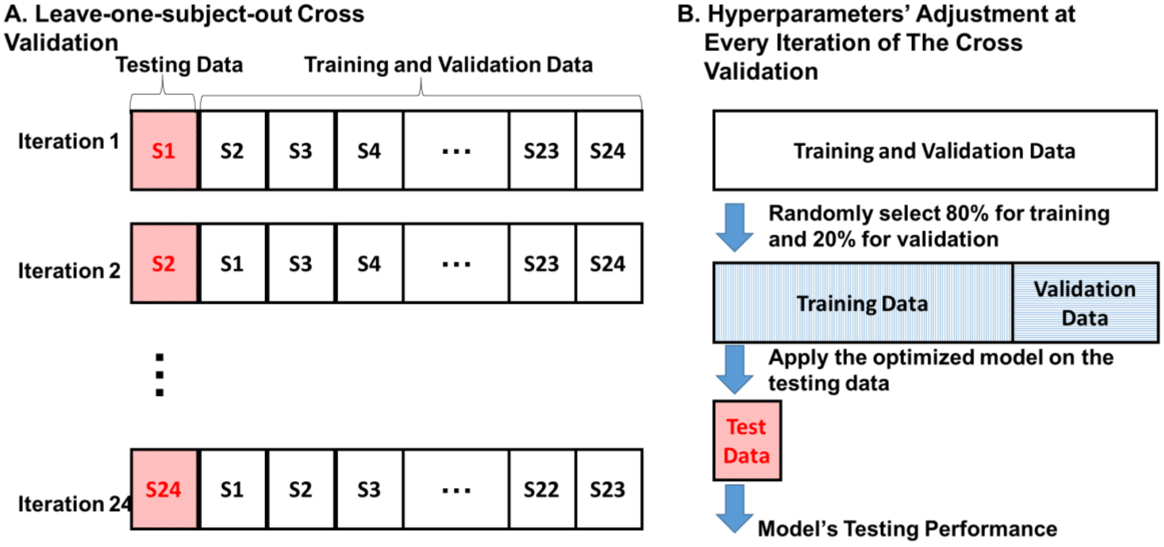

Figure S1 - **A.** Schematic of the leave-one-subject-out cross-validation. **B.** Data split for training a model and hyperparameters' adjustments at every iteration.

[1] Steyerberg, E.W., Harrell Jr, F.E., Borsboom, G.J., Eijkemans, M.J.C., Vergouwe, Y. and Habbema, J.D.F., 2001. Internal validation of predictive models: efficiency of some procedures for logistic regression analysis. Journal of clinical epidemiology, 54(8), pp.774-781.
